# Single-cell RNA sequencing-guided drug discovery to alleviate radiotherapy-induced esophageal toxicity

**DOI:** 10.1016/j.apsb.2025.12.038

**Published:** 2025-12-27

**Authors:** Wenling Tu, Hangfeng Liu, Hongyu Lin, Jinkang Zhang, Tang Feng, Zhenyu Ding, Qing Li, Yuhong Shi, Zehua Zhou, Shuyu Zhang

**Affiliations:** a The Second Affiliated Hospital of Chengdu Medical College, Nuclear Industry 416 Hospital, Chengdu 610051, China; b Laboratory of Radiation Medicine, West China School of Basic Medical Sciences & Forensic Medicine, Sichuan University, Chengdu 610041, China; c School of Bioscience and Technology, Chengdu Medical College, Chengdu 610500, China; d NHC Key Laboratory of Nuclear Technology Medical Transformation (Mianyang Central Hospital), Mianyang 621099, China; e Department of Biotherapy, Cancer Center, West China Hospital, Sichuan University, Chengdu 610041, China

**Keywords:** Radiotherapy-induced esophageal toxicity (RIET), Single-cell RNA sequencing (scRNA-Seq), Drug discovery, MAPK/NF-*κ*B pathway, AP-1 transcription factor complex, T-5224, Metformin, Pleiotrophin (PTN)

To the Editor,

Radiotherapy-induced esophageal toxicity (RIET) is a common dose-limiting complication in thoracic radiotherapy with limited treatment options. RIET is a multifactorial process involving diverse cell types within a complex tissue structure. Single-cell RNA sequencing (scRNA-Seq) offers high-resolution insights into tissue heterogeneity. It has been successfully applied to radiation-related injuries in the skin, brain, intestine, and lung^1^, revealing pathogenic mechanisms and laying a mechanistic foundation for targeted drug discovery^2^, ^3^, ^4^. However, no scRNA-Seq-based investigations of RIET have yet been reported. To fill this gap, we established RIET rat models using both single- and fractionated-irradiation protocols and generated the first single-cell transcriptomic atlas of RIET in rats. We identified nine major cell types exhibiting distinct transcriptional dynamics, and integrative analyses uncovered shared differentially expressed genes, signaling pathways, and cell–cell communication networks involved in RIET self-remodeling. Guided by these insights, we conducted mechanism-driven drug screening and identified three candidate agents with both radioprotective and radiosensitizing properties (Fig. 1A).

**Figure 1:**
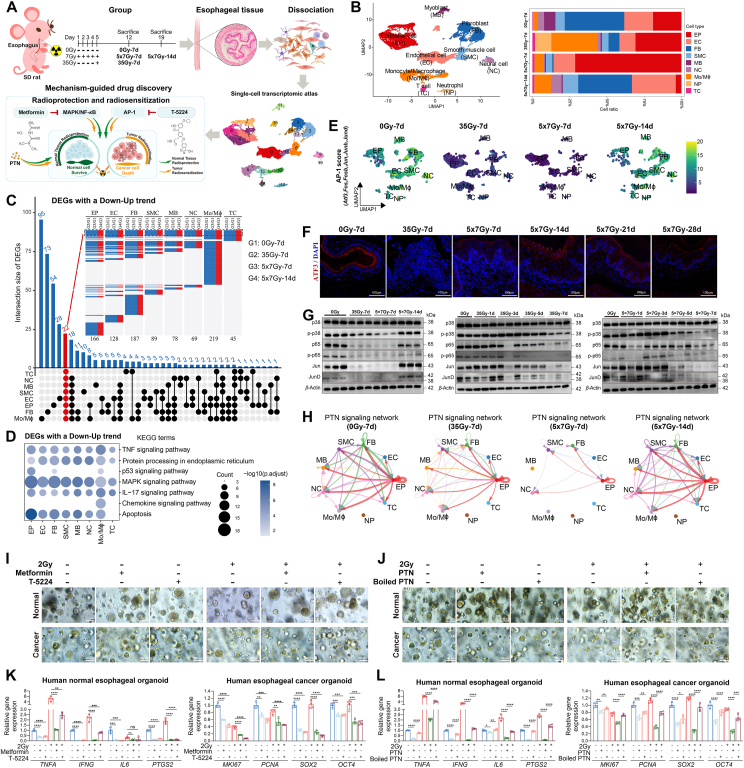
scRNA-Seq guides drug discovery to mitigate radiotherapy-induced esophageal toxicity. (A) Schematic overview of the experimental design. (B) UMAP plot of all cells colored by major cell types, alongside a bar plot displaying the relative proportions of each cell type across four groups. (C) Upset plot and heatmap showing unique and overlapping DEGs with a Down-Up trend. Red text highlights DEGs shared across most cell types. (D) KEGG enrichment of Down-Up DEGs. (E) UMAP plots showing AP-1 scores across four groups. (F) Immunofluorescence staining of Atf3 across six time points. Scale bar, 100 μm. (G) Western blot analysis of MAPK/NF-*κ*B/AP-1 axis proteins at selected time points post-irradiation. (H) Circle plots showing PTN signaling interactions across four groups. (I, J) Radioprotective and radiosensitizing effects of T-5224, metformin, and rhPTN in both human normal esophageal and esophageal cancer organoids. Scale bar, 100 μm. (K, L) mRNA levels of cytokines (*TNFA*, *IFNG*, *IL6*, and *PTGS2*) and stemness markers (*MKI67*, *PCNA*, *SOX2*, and *OCT4*). Data are represented as mean ± SD and analyzed by an unpaired 2-tailed *t*-test (K, L, *n* = 4). ∗*P* < 0.05; ∗∗*P* < 0.01; ∗∗∗*P* < 0.001; ∗∗∗∗*P* < 0.0001; ns, not significant.

## 1 Single-cell transcriptome profiling revealed cell type composition and distributional changes during RIET

Upon histopathological examination, esophageal tissues from non-irradiated controls (0Gy-7d) and irradiated rats (35Gy-7d, 5 × 7Gy-7d, 5 × 7Gy-14d, 5 × 7Gy-21d, and 5 × 7Gy-28d) showed epithelial thickening and inflammatory infiltration at 7 days post-radiation, with substantial recovery by 14 days, with minor differences in pathological manifestations at 14, 21, and 28 days post-radiation (Supporting Information Fig. S1A). To resolve the cellular and transcriptional heterogeneity underlying these changes, we generated single-cell atlases for 0Gy-7d, 35Gy-7d, 5 × 7Gy-7d, and 5 × 7Gy-14d groups. Nine major cell types were identified, including epithelial cell (EP), endothelial cell (EC), fibroblast (FB), smooth muscle cell (SMC), myoblast (MB), neural cell (NC), monocyte/macrophage (Mo/M*ɸ*), neutrophil (NP), and T cell (TC) (Fig. 1B and Fig. S1B–S1D). Early after irradiation, EP, Mo/M*ɸ*, NP, and TC populations expanded, whereas FB, EC, SMC, NC, and MB declined (Fig. 1B and Fig. S1E). By Day 14, cellular composition largely normalized, although EP abundance remained reduced (Fig. 1B and Fig. S1E). These dynamics suggest sequential activation of epithelial–mesenchymal transition (EMT) and mesenchymal–epithelial transition (MET) programs during tissue remodeling. Notably, the transient decrease and recovery of EMT score, together with its further elevation in FB at 5 × 7Gy-14d (Supporting Information Fig. S2A), indicate the onset of fibrosis. This was corroborated by polarized light imaging of picrosirius red-stained collagen, which revealed progressive collagen accumulation from Day 14 and markedly greater deposition by Day 28 (Fig. S2B). Collectively, these findings revealed dynamic shifts during self-remodeling following RIET.

## 2 Gene expression dynamics across cell types during RIET at single-cell resolution

To further elucidate molecular events at the cellular level, we compared gene expression patterns across groups: 35Gy-7d *vs* 0Gy-7d, 5 × 7Gy-7d *vs* 0Gy-7d, 5 × 7Gy-14d *vs* 0Gy-7d, and 5 × 7Gy-14d *vs* 5 × 7Gy-7d. Notably, the differentially expressed genes (DEGs) in 5 × 7Gy-14d *vs* 0Gy-7d were smaller than those of other comparisons (Supporting Information Fig. S3A), suggesting transcriptomic similarity between 5 × 7Gy-14d and 0Gy-7d to a certain extent. Integrating these findings with histopathological observations, cellular composition, and gene expression patterns, we hypothesized that a time-dependent transcriptional shift occurs during RIET, with early-phase dysregulation in 35Gy-7d and 5 × 7Gy-7d partially restored by 5 × 7Gy-14d. To test this, we classified DEGs into Down-Up and Up-Down trends (Fig. 1C and Fig. S3B). The Down-Up trend was characterized by initial downregulation in the 35Gy-7d and 5 × 7Gy-7d, followed by upregulation in the 5 × 7Gy-14d. The Up-Down trend followed the opposite pattern. KEGG pathway enrichment showed that DEGs with a Down-Up trend were mainly associated with MAPK signaling, TNF signaling, IL-17 signaling, apoptosis, and endoplasmic reticulum protein processing pathways (Fig. 1D). In contrast, DEGs with an Up-Down trend were mainly related to ribosome and oxidative phosphorylation (Fig. S3C). Interestingly, 22 shared DEGs exhibited a consistent Down-Up trend across most cell types (Fig. S3D). Among these, *Atf3*, *Fos*, *Fosb*, *Jun*, *Junb*, and *Jund*—subunits of the AP-1 transcription factor complex—were nearly undetectable at the 7th day post-radiation, but returned to basal levels by the 14th day in most cell types (Fig. 1E and Fig. S3D). Immunofluorescence confirmed these findings for ATF3 expression (Fig. 1F). Previous studies have demonstrated that TNF or IL-17 signals can trigger the activation of the MAPK signal that sustains AP-1 or NF-*κ*B activity^5^. Consistently, our gene set score analyses and WB experiments confirmed coordinated changes in MAPK, NF-*κ*B, and AP-1 signals in the 0Gy-7d, 35Gy-7d, 5 × 7Gy-7d, and 5 × 7Gy-14d groups (Supporting Information Fig. S4 and Fig. 1G). Moreover, we demonstrated that these signals exhibited similar dynamics at early time points after irradiation, being activated on the 1st day and gradually declining by the 5th day (Fig. 1G). Collectively, these results support the hypothesis that MAPK, NF-*κ*B, AP-1, and other related signals may play pivotal roles in orchestrating the self-remodeling process following RIET.

## 3 Dynamic remodeling of cell–cell communication networks during RIET

To explore intercellular communication mechanisms contributing to RIET, we performed Cell Chat analysis to evaluate the dynamic evolution of cell–cell interactions following irradiation. Based on differences in the overall information flow, pleiotrophin (PTN) signaling exhibited a unique pattern of downregulation in both 35Gy-7d and 5 × 7Gy-7d, followed by reactivation in 5 × 7Gy-14d (Supporting Information Fig. S5B). Within the PTN signaling network, EP assumed a predominant role (Fig. 1H and Fig. S5A–S5C). Furthermore, ligand–receptor pair analysis revealed that the cell–cell communication scores of Ptn-Sdc2 between EP (senders) and FB (receivers), and Ptn-Ncl between EP (senders or receivers) and other cell types (receivers or senders) followed the same Down-Up trend (Fig. S5D). These findings suggest that EP exhibits both cell-autonomous and non-cell-autonomous effects of PTN signaling within the RIEI microenvironment.

## 4 Targeting key signals for radioprotection and radiosensitization in the human esophagus based on scRNA-Seq

scRNA-Seq analysis revealed several shared molecular programs involved in RIET pathogenesis, highlighting the MAPK/NF-*κ*B/AP-1 axis and PTN signaling as potential druggable targets. To explore this possibility, we conducted a two-pronged investigation in human normal esophageal epithelial cell lines (Het-1A and HEEC) and esophageal cancer cells (KYSE150), followed by functional assessment in patient-derived normal esophageal and esophageal cancer organoids. The first step targeted AP-1, MAPK, NF-*κ*B, or oxidative phosphorylation using a panel of 11 pharmacological agents. Among these, the AP-1 inhibitor T-5224 and the MAPK/NF-*κ*B modulator metformin demonstrated relatively favorable cellular tolerability (Supporting Information Fig. S6). Pre-treatment with either T-5224 or metformin prior to irradiation significantly improved cellular viability, reduced reactive oxygen species (ROS) and lactate dehydrogenase (LDH) release, decreased apoptosis rates, and restored cell morphology, both with and without radiation exposure (Supporting Information Fig. S7A–S7E). In addition, both agents attenuated radiation-induced upregulation of inflammatory cytokines (*TNFA*, *IFNG*, *IL6*, and *PTGS2*) (Supporting Information Fig. S8A). Specifically, metformin effectively suppressed radiation-induced activation of the AMPK/MAPK/NF-*κ*B/AP-1 axis (Supporting Information Fig. S9). The second step focused on PTN signaling. Overexpression of PTN *via* an adenoviral vector significantly enhanced cellular radiation resistance, as evidenced by improved viability, reduced ROS levels, decreased LDH release, and preserved morphology (Fig. S7F–S7I). PTN also markedly decreased radiation-induced apoptosis in both Het-1A and HEEC cells (Fig. S7J). To assess potential differential effects on tumor *versus* normal cells, we also examined the impact of T-5224, metformin, and PTN overexpression on the radiosensitivity of esophageal cancer cells (KYSE150). Interestingly, all three treatments significantly reduced viability and increased apoptosis in KYSE150 cells (Fig. S8B and S8C), suggesting potential radiosensitizing effects in malignancy.

To extend these findings to a more physiologically relevant model, we further investigate the dual functions of T-5224, metformin, and recombinant human PTN (rhPTN) in both human normal esophageal and esophageal cancer organoids. Pre-treatment with either T-5224 or metformin enhanced organoid formation and growth in both irradiated and non-irradiated normal esophageal organoids (Fig. 1I), while attenuating the radiation-induced transcription of *TNFA*, *IFNG*, *IL6*, and *PTGS2* (Fig. 1K). In contrast, the same treatments led to the destruction of esophageal cancer organoids and intensified radiation-induced damage (Fig. 1I), accompanied by reduced expression of stem cell markers *MKI67*, *PCNA*, *SOX2*, and *OCT4* (Fig. 1K). Similarly, rhPTN exhibited analogous effects in both irradiated and non-irradiated human normal esophageal and esophageal cancer organoids (Fig. 1J and L). The heat-inactivated rhPTN eliminated these biological effects (Fig. 1J and L). Together, these findings demonstrate that T-5224, metformin, and rhPTN exert both radioprotective effects in the normal esophagus and radiosensitizing effects in esophageal cancer, underscoring their translational potential for optimizing clinical radiotherapy.

In summary, our study first established a comprehensive single-cell transcriptomic atlas of rat RIET and demonstrated the power of scRNA-Seq to guide mechanism-based drug discovery. We identified the MAPK/NF-*κ*B/AP-1 axis and PTN signaling as central regulators of radiation response. Pharmacological targeting of these pathways with T-5224, metformin, and rhPTN effectively mitigated RIET and sensitized esophageal cancer to radiotherapy, offering new opportunities to inform future radiotherapy regimen design.

## Author contributions

Wenling Tu analyzed the scRNA-Seq data. Wenling Tu, Hangfeng Liu, and Hongyu Lin contributed to the writing of the paper. Wenling Tu, Hangfeng Liu, and Jinkang Zhang performed all rat experiments. Hongyu Lin and Jinkang Zhang performed all cell experiments. Qing Li, Tang Feng and Zhenyu Ding performed all organoid experiments. Shuyu Zhang, Zehua Zhou, and Yuhong Shi designed the experiments, supervised the entire research process, and took responsibility for the study.

## Conflicts of interest

The authors declare no conflicts of interest.

## Appendix A Supporting information

The following is the Supporting Information to this article:
