# Supplementary material for: Single-cell RNA sequencing-guided drug discovery to alleviate radiotherapy-induced esophageal toxicity
Source: Acta Pharm Sin B. 2025 Dec 27;16(3):1769–72. doi: 10.1016/j.apsb.2025.12.038 (PMC13031072; doi:10.1016/j.apsb.2025.12.038)
Supplement: Multimedia component 1 [file mmc1.docx]

**Supporting Information for**

**Letter to the editor**

**Single-cell RNA sequencing-guided drug discovery to alleviate radiotherapy-induced esophageal toxicity**

**Wenling Tu^a,c,d,†^, Hangfeng Liu^a,†^, Hongyu Lin^a,c,†^, Jinkang Zhang^a,c^, Tang Feng^e^, Zhenyu Ding^e^, Qing Li^e^, Yuhong Shi^a,*^, Zehua Zhou^a,*^, Shuyu Zhang^a,b,d,*^**

^a^*The Second Affiliated Hospital of Chengdu Medical College, Nuclear Industry 416 Hospital, Chengdu 610051, China*

^b^*Laboratory of Radiation Medicine, West China School of Basic Medical Sciences & Forensic Medicine, Sichuan University, Chengdu 610041, China*

^c^*School of Bioscience and Technology, Chengdu Medical College, Chengdu 610500, China*

^d^*NHC Key Laboratory of Nuclear Technology Medical Transformation (Mianyang Central Hospital), Mianyang 621099, China*

^e^*Department of Biotherapy, Cancer Center, West China Hospital, Sichuan University, Chengdu 610041, China*

Received 29 July 2025; received in revised form 26 September 2025; accepted 16 October 2025

^†^These authors contributed equally to this work.

^*^Corresponding authors.

E-mail addresses: zhangshuyu@scu.edu.cn (Shuyu Zhang), 102024026@cmc.edu.cn (Zehua Zhou), shiyuhong@cmc.edu.cn (Yuhong Shi).

# 1. Supporting materials

## *1.1. Antibodies and chemical regents.*

| **Antibodies identifier** | **Source** | **(Cat number)** |
| --- | --- | --- |
| AMPK*α* | CST | 5831T |
| Phospho-AMPK*α* (Thr172) | CST | 2535T |
| NF-*κ*B p65 | CST | 8242S |
| Phospho-NF-*κ*B p65 (Ser536) | CST | 3033S |
| p38 MAPK | CST | 8690S |
| Phospho-p38 MAPK (Thr180/Tyr182) | CST | 4511T |
| c-Jun | CST | 9165T |
| JunD | Affinity | AF6200 |
| ATF3 | Affinity | DF3110 |
| *β*-Actin | Affinity | T0022 |
| Goat Anti-Rabbit IgG (H+L) HRP | Affinity | S0001 |
| Goat Anti-Mouse IgG (H+L) HRP | Affinity | S0002 |
| Donkey anti-Rabbit IgG (H+L) highly cross-adsorbed secondary antibody, Alexa Fluor 568 | Invitrogen | A10042 |
| **Reagent identifier** |  |  |
| Collagenase I | Gibco | #17018029 |
| DNase I | Applichem | #A3778.0050 |
| 0.125% trypsin-EDTA | Gibco | #15090046 |
| Dead cell removal Kit | MACS | #130-090-101 |
| BSA | MACS | #1000076 |
| Matrigel | bioGenous | M315066 |
| Human esophageal organoid kit | bioGenous | K2040-HES |
| Esophageal cancer organoid kit | bioGenous | K2177-ES |
| TrypLE^™^ Express | Gibco | 12605010 |
| human PTN recombinant protein | FineTest | P4903 |
| H&E staining Kit | Servicebio | G1076 |
| Picro Sirius Red solution | Servicebio | G1078 |
| Dulbecco’s modified Eagle’s medium (DMEM) | Gibco | C11995500BT |
| Roswell Park memorial institute 1640 medium (RPMI 1640) | Gibco | C11875500BT |
| Fetal bovine serum (FBS) | VivaCell | C04001-500 |
| Trypsin EDTA Solution A | VivaCell | C3530-0500 |
| TPA | Selleck Chemicals | S7791 |
| T-5224 | Selleck Chemicals | S8966 |
| Asiatic acid | Selleck Chemicals | S2266 |
| Anisomycin | Selleck Chemicals | S7409 |
| Metformin HCl | Selleck Chemicals | S1950 |
| SB202190 | Selleck Chemicals | S1077 |
| SB203580 | Selleck Chemicals | S1076 |
| Rotenone | Selleck Chemicals | S2348 |
| Coenzyme Q10 | Selleck Chemicals | S2398 |
| BAY 11-7082 | Selleck Chemicals | S2913 |
| Betulinic acid | Selleck Chemicals | S3603 |
| Cell Counting Kit-8 (CCK-8) | APExBIO | K1018 |
| LDH Cytotoxicity Assay Kit | Beyotime | C0017 |
| ROS Assay Kit with CM-H2DCFDA | Beyotime | S0035S |
| Annexin V-FITV/PI apoptosis detection Kit | YEASEN | 40302ES60 |
| Lysis buffer | Bioteke | PP1801 |
| Phenylmethylsulfonyl fluoride | Solarbio | P0100 |
| Protease inhibitor cocktail | MCE | HYK0010 |
| BCA protein assay Kit | CWBIO | CW0014S |
| Omin-EasyTMONE-STEP PAGE Gel Fast Preparation Kit | [Yamay](https://www.bing.com/ck/a?!&&p=5afb0ae1871bd6d4JmltdHM9MTcyMTE3NDQwMCZpZ3VpZD0xMGU3NzgzNi1lOWNkLTY2NzgtMjc2MC02OWU1ZThhYjY3M2ImaW5zaWQ9NTYzNg&ptn=3&ver=2&hsh=3&fclid=10e77836-e9cd-6678-2760-69e5e8ab673b&psq=%e9%9b%85%e9%85%b6%e5%85%ac%e5%8f%b8%e5%85%a8%e7%a7%b0%e8%8b%b1%e6%96%87&u=a1aHR0cHM6Ly93d3cucWNjLmNvbS9maXJtLzVkYjgyYjE4ZTc2YmE1ZWRkNTJmMmIyMGVlZTgwMDgzLmh0bWw&ntb=1) | PG212 |
| TRIzol | Thermo Fisher Scientific | 15596018CN |
| FastKing RT Kit (With gDNase) | TIANGEN | KR116 |
| SYBR^®^Green Real-time PCR Master Mix kit | TIANGEN | FP205 |
| PTN ELISA kit | FineTest | EH1480 |
| human recombinant PTN | FineTest | P4903 |

## *1.2. Adenovirus and infection*

To overexpress human PTN in Het-1A, HEEC and KYSE150 cells, PTN overexpressing adenovirus (Ad-PTN) and the negative control adenovirus (Ad-NC) were obtained from Hanbio Biotechnology Co., Ltd. (Shanghai, China). The infection was performed according to the standard procedures.

# 2. Supporting methods

## *2.1. Animal models*

All animal procedures were approved by the Animal Experimentation Ethics Committee of the Second Affiliated Hospital of Chengdu Medical College, China National Nuclear Corporation 416 Hospital (permit no. DWSB-2024-051). Male Sprague–Dawley (SD) rats (6 weeks old) were purchased from Chengdu Dossy Experimental Animals Co., Ltd. (Chengdu, China). Thirty rats were randomly assigned to six groups (0Gy-7d, 35Gy-7d, 5×7Gy-7d, 5×7Gy-14d, 5×7Gy-21d, and 5×7Gy-28d; *n* = 5 per group), and another twenty seven rats were assigned to nine groups (0Gy-1d, 5×7Gy-1d, 5×7Gy-3d, 5×7Gy-5d, 5×7Gy-7d, 35Gy-1d, 35Gy-3d, 35Gy-5d, and 35Gy-7d; *n* = 3 per group). For stable irradiation, rats were anesthetized with intraperitoneal injections of ketamine (75 mg/kg) and xylazine (10 mg/kg). Fur in the cervicothoracic region was shaved, and a 3 cm × 4 cm esophageal target field was defined below the ear. Other body regions were shielded with 3-cm-thick lead plates. Radiation doses of 5 × 0 Gy, 1 × 35 Gy, or 5 × 7 Gy (7 Gy administered daily for 5 days) were administered to the esophageal region at a fixed dose rate of 1.7 Gy/min using an X-ray linear accelerator (model XCELL 320; KUBTEC Scientific, Milford, CT, USA). Rats were sacrificed at 1, 3, 5, 7, 14, 21 or 28 days post-radiation, and esophageal tissues were collected for scRNA-Seq, Hematoxylin and Eosin (H&E) staining, Sirius Red staining, immunofluorescence, or Western blotting.

## *2.2.* *Hematoxylin and Eosin (H&E) staining, Sirus Red staining and tissue immunofluorescence*

Rat esophageal tissues were fixed in 4% paraformaldehyde, paraffin-embedded, and sectioned for staining. Sections were deparaffinized, rehydrated, and subjected to H&E staining (H&E Staining Kit, G1076; Servicebio, Wuhan, China), and Sirius Red staining (Picro Sirius Red solution, G1078; Servicebio, Wuhan, China). For immunofluorescence, 10-μm sections were blocked, incubated with anti-ATF3 antibodies (DF3110; Affinity, Cincinnati, OH, USA) and corresponding red-fluorescent secondary antibodies (A10042; Invitrogen, Waltham, MA, USA), counterstained with nuclear DAPI, and mounted with fluorescent medium. H&E and immunofluorescence images were captured using a digital slide scanner (Pannoramic MIDI; 3DHISTECH Ltd., Budapest, Hungary), while Sirius Red images were acquired using a polarized light microscope (Eclipse ci; Nikon Instruments, Tokyo, Japan).

## *2.3. Single-cell RNA sequencing (scRNA-Seq)*

Fresh rat esophageal tissues from 0Gy-7d, 35Gy-7d, 5×7Gy-7d and 5×7Gy-14d groups were stored in MACS tissue storage solution (#130-100-008; Miltenyi, Bergisch Gladbach, Germany) at 4 °C and shipped to the processing lab within 48 h for scRNA-Seq. Esophageal tissues from five rats per group were pooled for processing. Each pooled sample was finely chopped and enzymatically digested with 5 mg/mL collagenase I (#17018029; Gibco, Waltham, MA, USA), 1 mg/mL DNase I (#A3778.0050; Applichem, Darmstadt, Germany), and 0.125% trypsin-EDTA (#15090046; Gibco, Waltham, MA, USA) in a shaker at 37 °C for 60 min. The resulting cell suspension was filtered through a 40 μm strainer (#352340; Falcon, NY, USA) and sorted using a Dead Cell Removal Kit (#130-090-101; MACS, Bergisch Gladbach, Germany) to eliminate dead cells. The cells were then resuspended in D-PBS containing 0.04% BSA (#1000076; MACS, Bergisch Gladbach, Germany) at a concentration of approximately 1000 cells/μL in preparation for scRNA-Seq. scRNA-Seq libraries were prepared using the Chromium Single Cell 3′ Library & Gel Bead Kit v3.1 (10x Genomics) following the manufacturer’s protocol. The final libraries were sequenced using an Illumina NovaSeq 6000 sequencer. scRNA-Seq was performed by OE Biotech (Shanghai, China).

## *2.4.* *Quality control and preprocessing of scRNA-Seq data*

Raw gene expression matrices were generated for each sample using the Cell Ranger Pipeline (version 5.0.0) coupled with the mRatBN7.2/rn7 rat reference. Seurat R package (version 4.0.0) was employed to create a Seurat object from the gene expression matrix of each sample, process each object, and subsequently integrate them. Cells were filtered based on the following criteria: nFeature_RNA  >  200 & nFeature_RNA  < 5000 & percent.mito < 15. Doublets were detected and removed using the DoubletFinder R package (version 2.0.3). After stringent cell filtration, a total of 28,804 qualified cells (10,713 from 0Gy-7d, 8,771 from 35Gy-7d, 8,523 from 5x7Gy-7d, 9,568 from 5x7Gy-14d) were retained for subsequent analyses.

## *2.5.* *Cell clustering and identification analyses*

The merged Seurat objects were further processed using the Seurat functions. Data normalization was performed with the NormalizeData function, followed by feature selection using FindVariableFeatures with 2000 features. Data scaling was conducted using ScaleData, regressing out cell cycle genes, number of UMIs, and mitochondrial genes. Principal component analysis (PCA) and uniform manifold approximation and projection (UMAP) were then applied for dimensional reduction, utilizing the top 50 principal components (RunPCA and RunUMAP). Nearest neighbor graphs were constructed based on these principal components using FindNeighbors. Cell clusters were identified using FindClusters with a resolution parameter of 0.5, based on the nearest neighbor graph. Cell-type labels were assigned to each cluster based on known marker genes. This procedure yielded nine cell type-specific clusters representing epithelial cell, endothelial cell, fibroblast, smooth muscle cell, myoblast, neural cell, monocyte/macrophage, neutrophil, and T cell. The Seurat function FindAllMarkers (test.use = wilcox; min.pct = 0.25; logfc.threshold = 0.25, *P*_val_adj < 0.01) was used to identify differentially expressed genes (DEGs) by comparing cells from a meta-cluster to all cells from other meta-clusters.

## *2.6. Identification of DEGs with a Down-Up or Up-Down trend*

DEGs exhibiting a Down-Up or Up-Down trend were defined as follows: the Down-Up trend was characterized by initial downregulation in the 35Gy-7d and 5×7Gy-7d groups, followed by upregulation in the 5×7Gy-14d group. Conversely, the Up-Down trend showed initial upregulation in the 35Gy-7d and 5×7Gy-7d groups, with subsequent downregulation in the 5×7Gy-14d group. To identify DEGs with these trends, we firstly utilized the Seurat function FindAllMarkers to identify DEGs for the four comparisons of 35Gy-7d *vs* 0Gy-7d, 5×7Gy-7d *vs* 0Gy-7d, 5×7Gy-14d *vs* 0Gy-7d, and 5×7Gy-14d *vs* 5×7Gy-7d for each cell type. DEGs that were upregulated in 35Gy-7d *vs* 0Gy-7d and 5×7Gy-7d *vs* 0Gy-7d but downregulated in 5×7Gy-14d *vs* 5×7Gy-7d were classified as having an Up-Down trend. Conversely, DEGs that were downregulated in 35Gy-7d *vs* 0Gy-7d and 5×7Gy-7d *vs* 0Gy-7d but upregulated in 5×7Gy-14d *vs* 5×7Gy-7d were classified as having a Down-Up trend.

## *2.7. Gene function enrichment analyses*

Kyoto Encyclopedia of Genes and Genomes (KEGG) enrichment analyses were performed by the clusterProfiler R package (version 4.10.0). The top ranked KEGG terms (*P*_val_adj < 0.01) were visualized with the ggplot2 R package (Version 3.4.2).

## *2.8.* *Gene set score analyses*

The Seurat function AddModuleScore was used with default parameters to calculate the target gene set score for each cell. The gene set for MAPK signal score was obtained from the MSigDB KEGG database. Gene sets for NF-*κ*B signal score and epithelial-to-mesenchymal transition (EMT) score were obtained from the MSigDB HALLMARK database. The gene set for the AP-1 score included *Atf3*, *Fos*, *Fosb*, *Jun*, *Junb* and *Jund*.

## *2.9. Cell–cell communication analyses*

CellChat R package (Version 1.6.1) was used to perform cellular communication analysis with default setting, employing the “CellChatDB.mouse” ligand–receptor interaction database. Gene names were converted from rat to mouse using the biomaRt R package (Version 2.58.2). First, to identify potential interactions, we pre-processed the expression matrix using the in-built functions identifyOverExpressedGenes, identifyOverExpressedInteractions, and projectData. Next, we used the functions computeCommunProb, computeCommunProbPathway, and aggregateNet to infer the communication network and calculate communication probabilities. Finally, we visualized cell-to-cell connections, outgoing and incoming signaling patterns, specific signaling networks, and receptor–ligand interactions between specific cell compartments using the functions netVisual_circle, netAnalysis_signalingRole_heatmap, netVisual_heatmap, and netVisual_bubble.

## *2.10. Cell culture and irradiation*

Human normal esophageal epithelial cell lines Het-1A and HEEC, and cancer cell line KYSE150 were obtained from ATCC (Manassas, VA, USA) and ScienCell (Carlsbad, CA, USA). Cells were cultured in DMEM or RPMI-1640 (Gibco, Waltham, MA, USA) with 10% FBS (VivaCell, Shanghai, China) at 37 °C with 5% CO₂. For irradiation, cells were exposed to 0 or 8 Gy (1.7 Gy/min) using the KUBTEC XCELL 320. Cells were pretreated with metformin HCl Cells were pretreated with metformin HCl (200 μmol/L, Selleck Chemicals, Houston, TX, USA), T-5224 (30 or 50 μmol/L, Selleck Chemicals, Houston, TX, USA), or PTN-overexpressing adenovirus (Hanbio Biotechnology Co., Ltd., Shanghai, China) 24 h before radiation. Following radiation, cell viability, lactate dehydrogenase (LDH) release, ROS, apoptosis, and RT-qPCR assays were performed, with experimental details provided in the Supporting materials and methods.

## *2.11. Agonists and inhibitors*

To screen out which drugs may be used to alleviate RIEI, Het-1A and HEEC cells were used to screened 11 agonists and inhibitors targeting AP-1, MAPK, NF-*κ*B or oxidative phosphorylation. The agonists and inhibitors used in this study are as following: TPA (S7791; Selleck Chemicals, Houston, TX, USA), T-5224 (S8966; Selleck Chemicals, Houston, TX, USA), Asiatic acid (S2266; Selleck Chemicals, Houston, TX, USA), Anisomycin (S7409; Selleck Chemicals, Houston, TX, USA), Metformin HCl (S1950; Selleck Chemicals, Houston, TX, USA), SB202190 (S1077; Selleck Chemicals, Houston, TX, USA), SB203580 (S1076; Selleck Chemicals, Houston, TX, USA), Rotenone (S2348; Selleck Chemicals, Houston, TX, USA), Coenzyme Q10 (S2398; Selleck Chemicals, Houston, TX, USA), BAY 11-7082 (S2913; Selleck Chemicals, Houston, TX, USA), and Betulinic acid (S3603; Sellesck Chemicals, Houston, TX, USA). Ultimately, T-5224 and Metformin HCl were selected for subsequent experiments.

## *2.12.* *Cell viability assay*

Het-1A, HEEC and KYSE150 cells were digested with 0.25% trypsin at the logarithmic growth stage and then diluted into 5 × 10^3^ cells per well in 96-well plates, and then cells were treated with different drugs (200 µmol/L Metformin HCl, 30 or 50 µmol/L T-5224) or PTN overexpression adenovirus for 24 h before radiation exposure (0 and 8Gy). *In vitro* cell viability was measured using the Cell Counting Kit-8 (CCK-8) (K1018; APExBIO, Houston, TX, USA). Optical density was measured at 450 nm using a micro-plate reader (Biotek, Winooski, VT, Santa Clara, CA, USA).

## *2.13.* *Lactate dehydrogenase (LDH) release assay*

Het-1A and HEEC cells were pre-treated with indicated agents and exposed to X-ray irradiation (0 and 8 Gy). After drug stimulation, the cell culture plates were centrifuged by a perforated plate centrifuge 400 × *g* (L‑CFM‑2500, Shanghai Kehuai Instrument Co., Ltd., Shanghai, China) for 5 min. The supernatant was removed and 150 μL of LDH release reagent provided in the kit diluted 10-fold with PBS was added, and the plate was mixed well with appropriate shaking, and then continued to be incubated for 1 h in the cell culture incubator. Subsequently, the plates were centrifuged at 400 × *g* for 5 min, and 120 μL of supernatant from each well was added to the corresponding well of a new 96-well plate, and the samples were then measured at 490 nm according to the LDH assay kit (C0017, Beyotime, Shanghai, China).

## *2.14.* *Cellular reactive oxygen species (ROS) assay*

Het-1A and HEEC cells were pre-treated with indicated agents and exposed to X-ray irradiation (0 and 8 Gy). The levels of ROS were determined using the ROS sensitive dye 2′,7′-dichlorofluorescein diacetate (DCF-DA) (S0035S; Beyotime, Shanghai, China), which is converted by ROS into the highly fluorescent 2′,7′-dichlorofluorescein (DCF). The cells were incubated with 10 mmol/L DCF-DA for 20 min at 37 °C and subsequently washed with phosphate buffer saline (PBS). The level of ROS was performed using FACS Celesta flow cytometer (BectonDickinson, Franklin Lakes, NJ, USA). FlowJoTM (Version 10.7) was used to analyze the data.

## *2.15.* *Cell apoptosis assay*

Het-1A, HEEC and KYSE150 cells were pre-treated with indicated agents and exposed to X-ray irradiation (0 and 8 Gy). Then different assay kits were adopted according to the manuscript respectively, to evaluate the state of irradiated cells. Intensity of Annexin V/PI staining (40302ES60; Yesen, Shanghai, China) was monitored using a flowcytometry (BD Biosciences, San Jose, CA, USA). FlowJoTM (Version 10.7) was used to analyze data.

## *2.16.* *Western blotting (WB) analysis*

Protein was extracted using lysis buffer (PP1801, BioTeke, Beijing, China) supplemented with 1 mmol/L phenylmethylsulfonyl fluoride (P0100; Solarbio, Beijing, China) and 1 mmol/L protease Inhibitor Cocktail (HY-K0010; MCE, NJ, USA). The extracted protein solution was subjected to testing using a BCA kit and denatured with SDS. Subsequently, the proteins were separated at a constant voltage of 80 V for 40 min and 120 V for 60 min by Omin-Easy^™^ONE-STEP PAGE Gel Fast Preparation Kit (PG212; [Yamay](https://www.bing.com/ck/a?!&&p=5afb0ae1871bd6d4JmltdHM9MTcyMTE3NDQwMCZpZ3VpZD0xMGU3NzgzNi1lOWNkLTY2NzgtMjc2MC02OWU1ZThhYjY3M2ImaW5zaWQ9NTYzNg&ptn=3&ver=2&hsh=3&fclid=10e77836-e9cd-6678-2760-69e5e8ab673b&psq=%e9%9b%85%e9%85%b6%e5%85%ac%e5%8f%b8%e5%85%a8%e7%a7%b0%e8%8b%b1%e6%96%87&u=a1aHR0cHM6Ly93d3cucWNjLmNvbS9maXJtLzVkYjgyYjE4ZTc2YmE1ZWRkNTJmMmIyMGVlZTgwMDgzLmh0bWw&ntb=1), Shanghai, China) and transferred onto a PVDF membrane (Mannheim, Germany). After sealing with 5% skim milk powder, the primary antibodies against AMPK*α* (5831T; CST, Danvers, MA, USA), Phospho-AMPK*α* (2535T; CST, Danvers, MA, USA), p38 MAPK (8690S; CST, Danvers, MA, USA), Phospho-p38 MAPK (4511S; CST, Danvers, MA, USA), NF-*κ*B p65 (8242S; CST, Danvers, MA, USA), Phospho-NF-*κ*B p65 (3033S; CST, Danvers, MA, USA), c-Jun (9165T; CST, Danvers, MA, USA), JunD (AF6200; Affinity, Cincinnati, OH, USA), *β*-actin (T0022; Affinity, Cincinnati, OH, USA) were added overnight at 4 °C. After cleaning with TBST, the corresponding anti-rabbit (S0001; Affinity, Cincinnati, OH, USA) or anti-mouse (S0002; Affinity, Cincinnati, OH, USA) secondary antibody was added and incubated at 37 °C for 1 h, followed by washing with TBST and TBS. Then protein bands were visualized and photographed using a FluroChem M imaging system (Shenhua, Hangzhou, China).

## *2.17. Human normal esophageal and esophageal cancer organoid culture and irradiation*

Human esophageal cancer and paracancerous normal tissues were obtained from West China Hospital of Sichuan University, with ethical approval of the Ethics Committee at West China Hospital of Sichuan University (permit No. 2023 (789)) and informed consent from all patients. Single-cell suspensions were embedded in cold Matrigel (M315066; bioGenous, Shanghai, China) and seeded at 2 × 10⁴ cells per 50  μL per well in 24-well plates. After Matrigel polymerization at 37 °C, organoid media (bioGenous kits K2040-HES for normal and K2177-ES for cancer) were added. For passaging, organoids were dissociated using cold PBS and TrypLE^™^ Express (12605010; Gibco, Waltham, MA, USA) with gentle pipetting. After re-seeding, organoids were exposed to 0 or 2 Gy ionizing radiation at a fixed dose rate of 1.7 Gy/min.

Drug treatments included metformin HCl (100 µmol/L), T-5224 (10 µmol/L) and human recombinant PTN (100 ng/mL, P4903, FineTest, Wuhan, China), which were added 24  h before irradiation. Culture media were refreshed every 3 days. Post-radiation, images were taken, and reverse transcription quantitative polymerase chain reaction (RT-qPCR) were conducted.

## *2.18.* *Reverse transcription quantitative polymerase chain reaction analysis (RT-qPCR)*

RNA was extracted from human normal esophageal epithelial cell lines (Het-1A and HEEC), human normal esophageal organoid and human esophageal cancer organoid using TRIzol (15596018CN; Thermo Fisher Scientific, Waltham, MA, USA). The concentration and purity of RNA were assessed using a micronucleic acid protein quantifier (NanoDr0P One, Thermo Fisher Scientific, Waltham, MA, USA). The assay was performed and amplified according to the SYBR^®^Green Real-time PCR Master Mix kit (FP205; TIANGEN, Beijing, China) instructions. The period threshold (CT) was determined using quantitative PCR. The primer sequences are shown in Table S1.

**Table S1** The primer sequences.

| mRNA | 5′ to 3′ forward | 5′ to 3′ reverse |
| --- | --- | --- |
| *TNFA* | GCCTCGCCCTTTGCTTTACT | CTGTGGGTCTCAGGGAGATCA |
| *IFNF* | TCGGTAACTGACTTGAATGTCCA | TCGCTTCCCTGTTTTAGCTGC |
| *IL6* | ACTCACCTCTTCAGAACGAATTG | CCATCTTTGGAAGGTTCAGGTTG |
| *PTGS2* | CTGGCGCTCAGCCATACAG | CGCACTTATACTGGTCAAATCCC |
| *MKI67* | GTGGTTCGACAAGTGGCCT | ACAACAGGAAGCTGGATACGG |
| *SOX2* | CCAGCTCGCAGACCTACAT | GGAGGAAGAGGTAACCACA |
| *OCT4* | GGTATTCAGCCAAACGAC | CTTCCTCCACCCACTTCT |
| *PCNA* | AAAGCCACTCCACTCTCTTCAAC | TCCTTCTTCATCCTCGATCTTGG |
| *GAPDH* | TCCTCCACCTTTGACGCT | CCACCACCCTGTTGCTGT |

## *2.19. Statistics*

For the experimental data, GraphPad Prism 8.0 was used to perform statistical analyses and graphics production. An unpaired 2-tailed *t*-test was used to compare the means of 2 populations. * *P* < 0.05; ** *P* < 0.01; *** *P* < 0.001; **** *P* < 0.0001; ns, not significant.

**Other details**

Detailed descriptions of the methods and other methods used in this study can be obtained through e-mail contact with the corresponding author.

# 3. Supporting figures


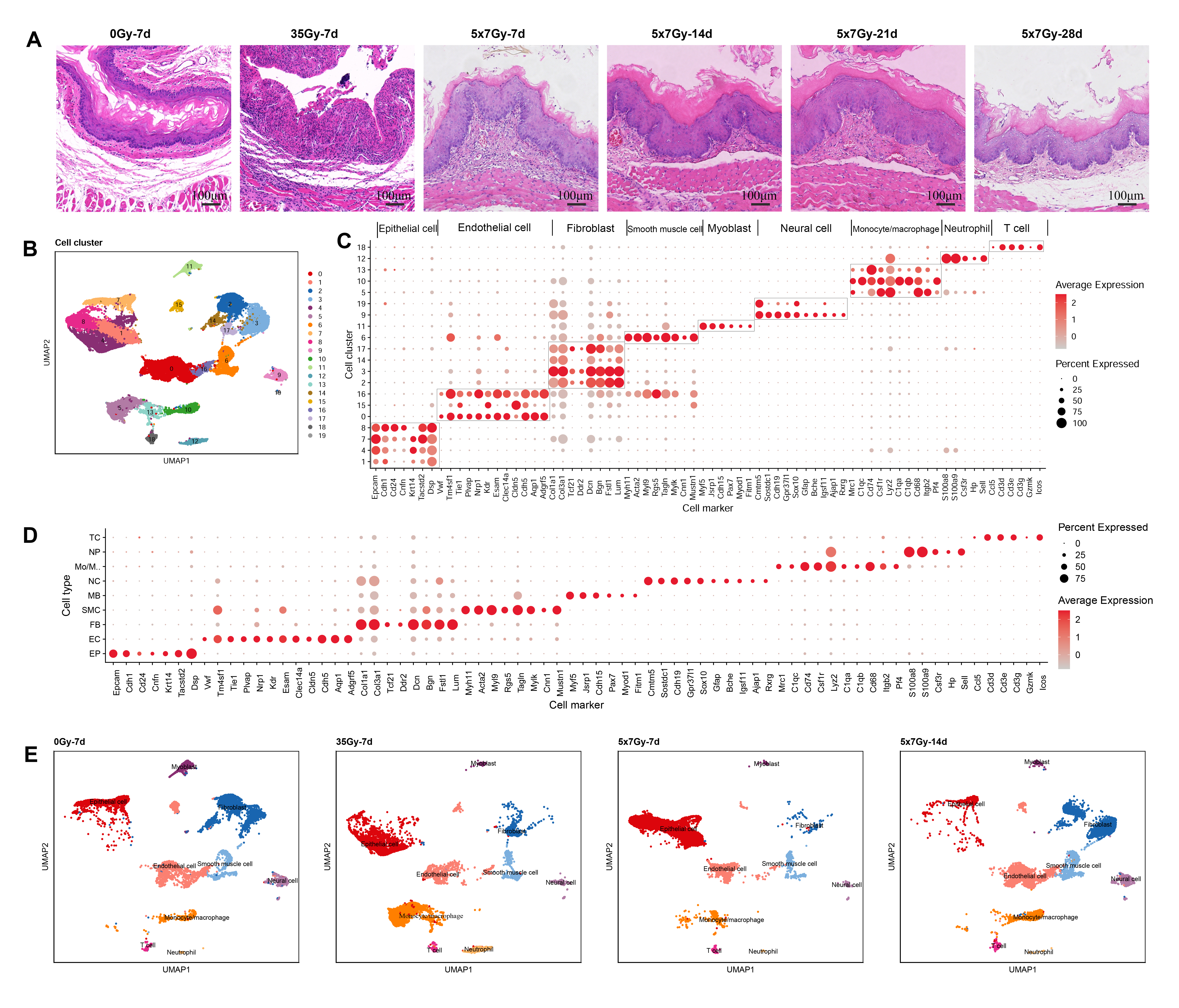


**Figure S1** Identification and distribution dynamics of cell types in radiotherapy-induced esophageal toxicity (RIET) *via* scRNA-Seq. (A) H&E staining of rat esophageal tissues from 0Gy-7d, 35Gy-7d, 5×7Gy-7d, 5×7Gy-14d, 5×7Gy-21d, and 5×7Gy-28d groups. (B) UMAP plot of all esophageal cells from four groups (0Gy-7d, 35Gy-7d, 5×7Gy-7d, and 5×7Gy-14d), colored by Seurat clusters. (C) Dot plot showing marker genes used to define each Seurat cluster. (D) Dot plot showing marker genes used to define each cell type. (E) Separate UMAP plots for each group.


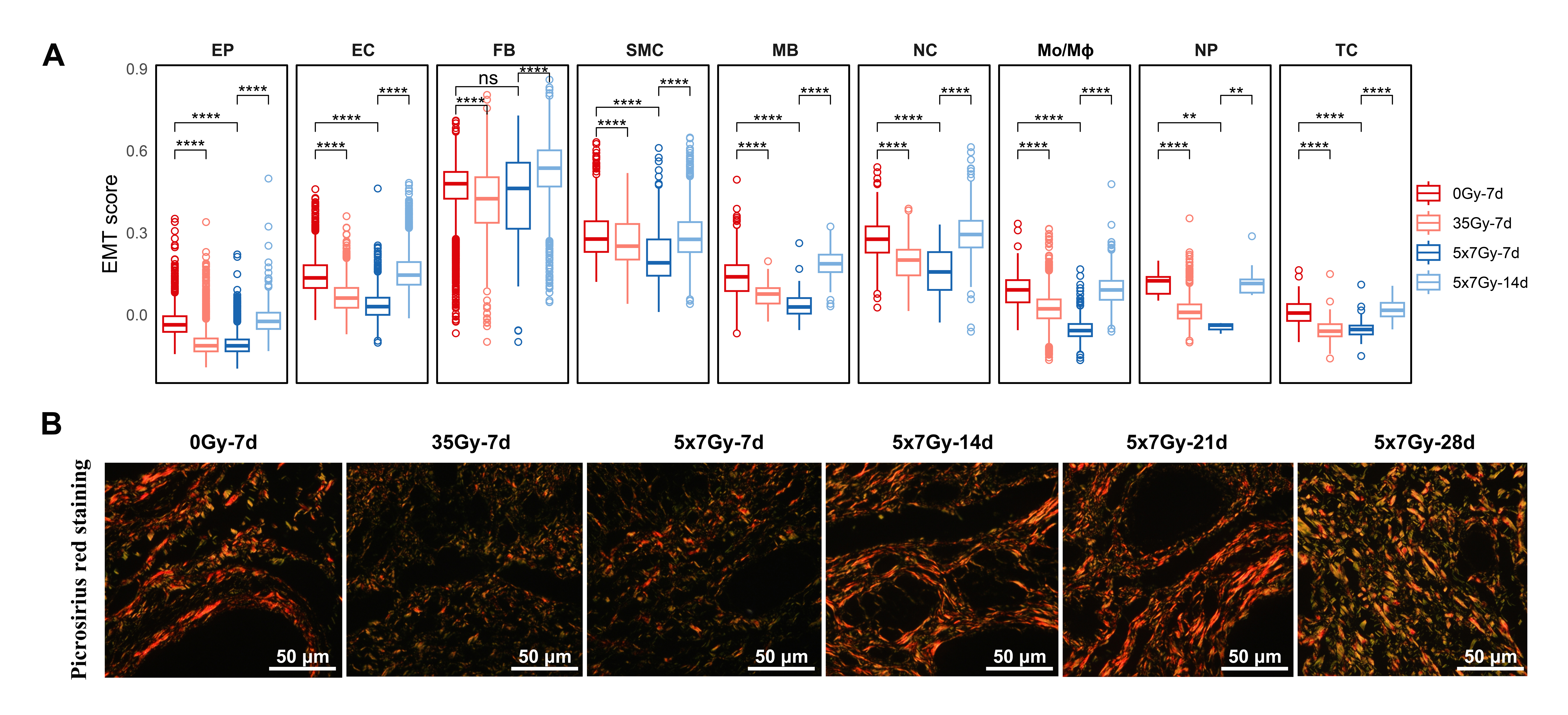


**Figure S2** Fibrotic changes during RIET. (A) Gene set score analysis of epithelial-to-mesenchymal transition (EMT) in various cell types of different groups. **P* < 0.05; ***P* < 0.01; *** *P* < 0.001; **** *P* < 0.0001; ns, not significant. (B) Representative picrosirius red staining of rat esophagus of 0Gy-7d, 35Gy-7d, 5x7Gy-7d, 5x7Gy-14d, 5x7Gy-21d and 5x7Gy-28d groups.

**

**

**Figure S3** Transcriptional dynamics across cell types during RIET. (A) Bar graphs showing the number of DEGs in four intergroup comparisons. (B) Upset plot and heatmap showing unique and overlapping DEGs with the Up-Down expression pattern. (C) KEGG enrichment of Up-Down DEGs. (D) Violin plots showing expression of shared Down-Up DEGs across cell types and groups. AP-1 subunits are highlighted in red.


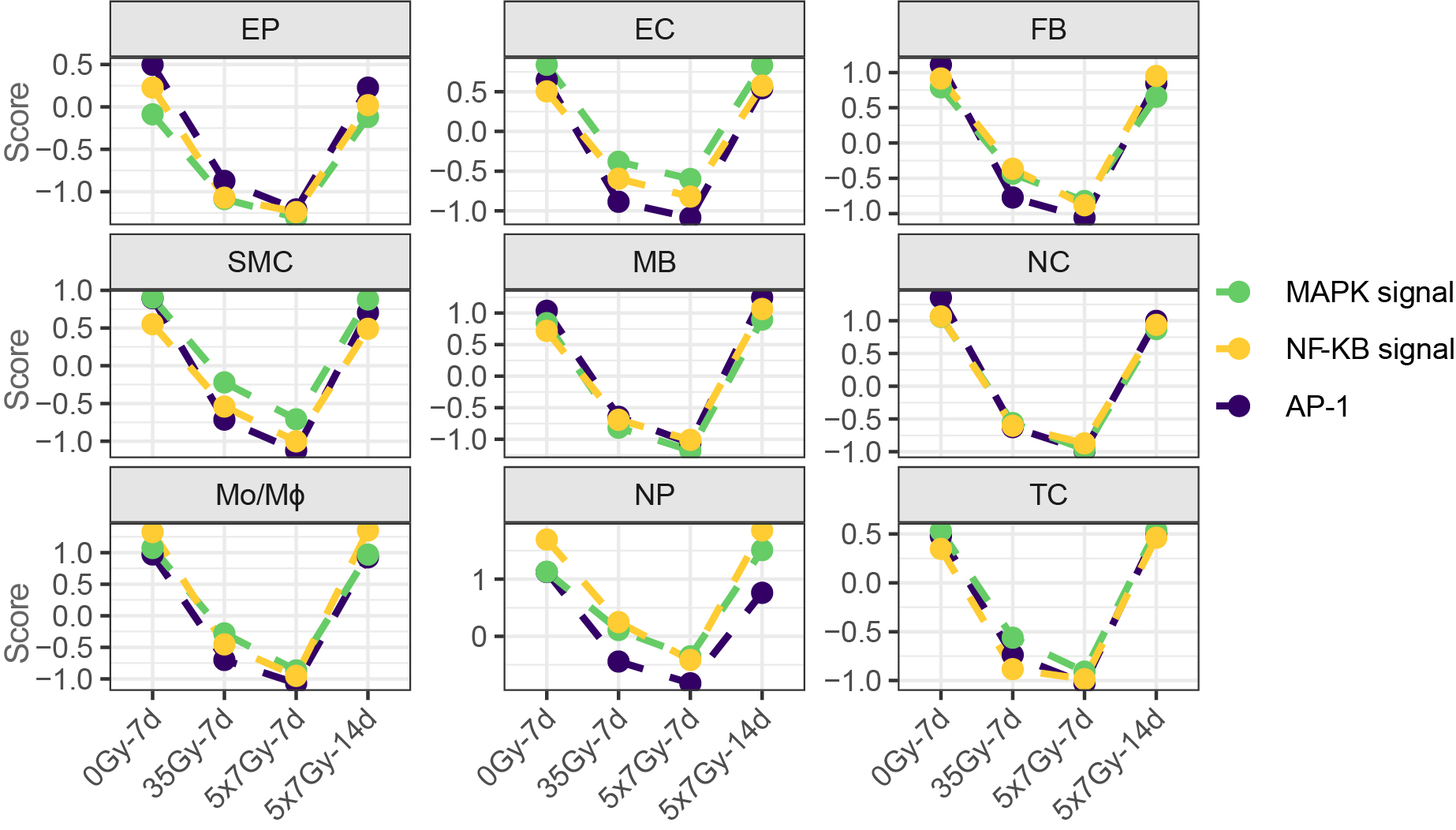


**Figure S4** The dynamics of MAPK, NF-*κ*B and AP-1 signals across cell types during RIET. Gene set score analyses of MAPK, NF-*κ*B and AP-1 signals were performed in various cell types of different groups.





**Figure S5** Cell–cell communication dynamics of esophageal microenvironment during RIET. (A) Heatmap shows outgoing and incoming signaling patterns of rat esophageal tissue. (B) Significant signaling pathways based on changes in information flow. (C) Heatmap of cell types in PTN signaling network. (D) Ligand–receptor pair analysis of PTN signaling between epithelial cells and other cell types across groups.


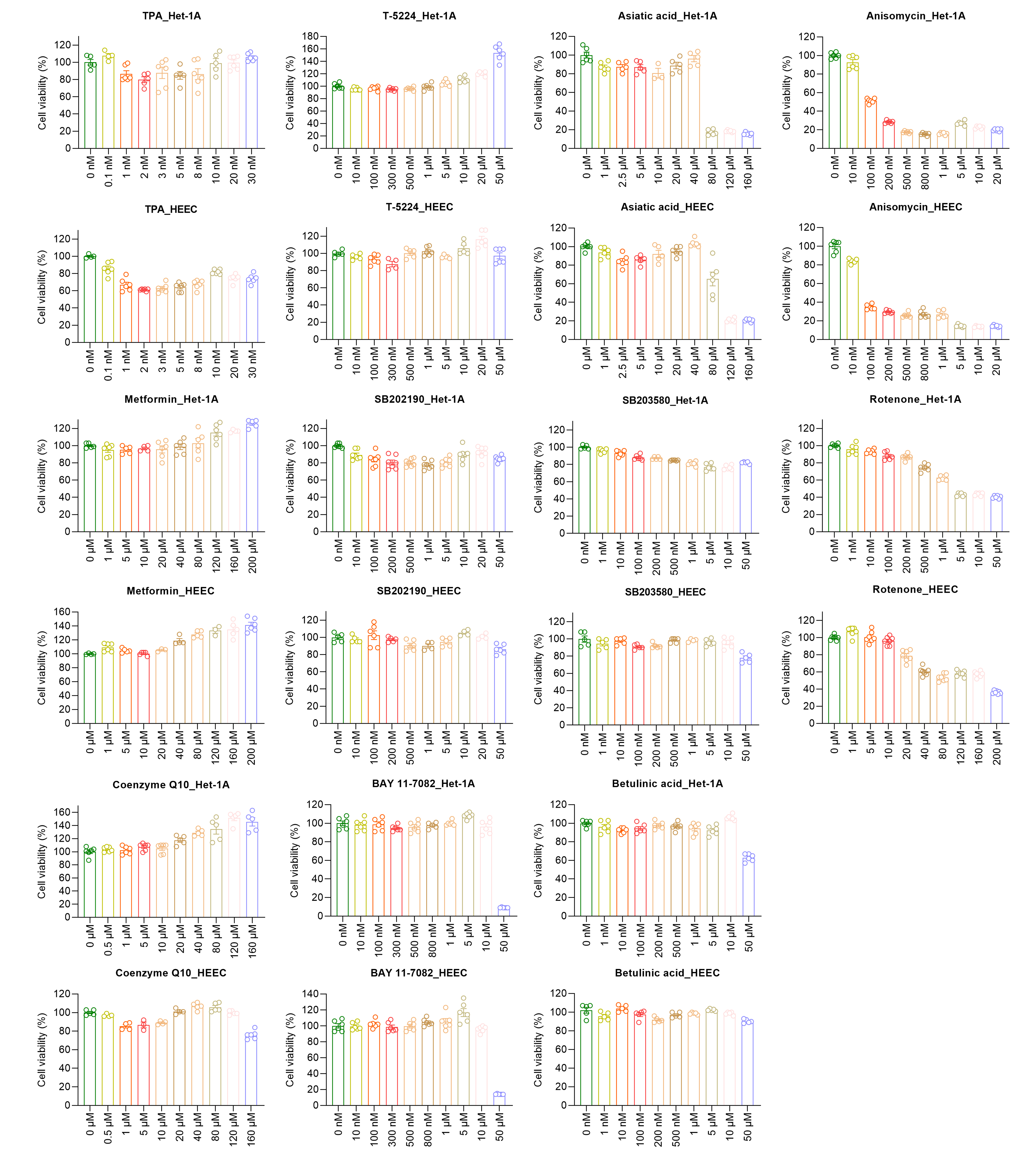


**Figure S6** Dose-dependent cytotoxicity of 11 candidate compounds in HEEC and Het-1A cells. Evaluation of cell viability following treatment with TPA (AP-1 agonist), T-5224 (AP-1 inhibitor), asiatic acid (MAPK agonist), anisomycin (MAPK agonist), metformin HCl (MAPK/NF-*κ*B modulator), SB202190 (MAPK inhibitor), SB203580 (MAPK inhibitor), rotenone (oxidative phosphorylation inhibitor), coenzyme Q10 (oxidative phosphorylation agonist), BAY 11-7082 (NF-*κ*B inhibitor) and betulinic acid (NF-*κ*B agonist).





**Figure S7** Radioprotective effects of T-5224, metformin, and PTN in human esophageal epithelial cells. (A–D) Assays of cell viability, LDH release, ROS, and apoptosis in irradiated/non-irradiated HEEC and Het-1A cells treated with T-5224 or metformin. (E, F) Bright-field images of treated HEEC/Het-1A cells or cells transduced with Ad-PTN or Ad-NC. (G–J) Corresponding functional assays in PTN-overexpressing cells. Data are presented as mean ± SD and analyzed by an unpaired 2-tailed *t*-test (A, B, C, D, G, H, I, J, *n* = 4). * *P* < 0.05; ** *P* < 0.01; *** *P* < 0.001; **** *P* < 0.0001; ns, not significant.

**
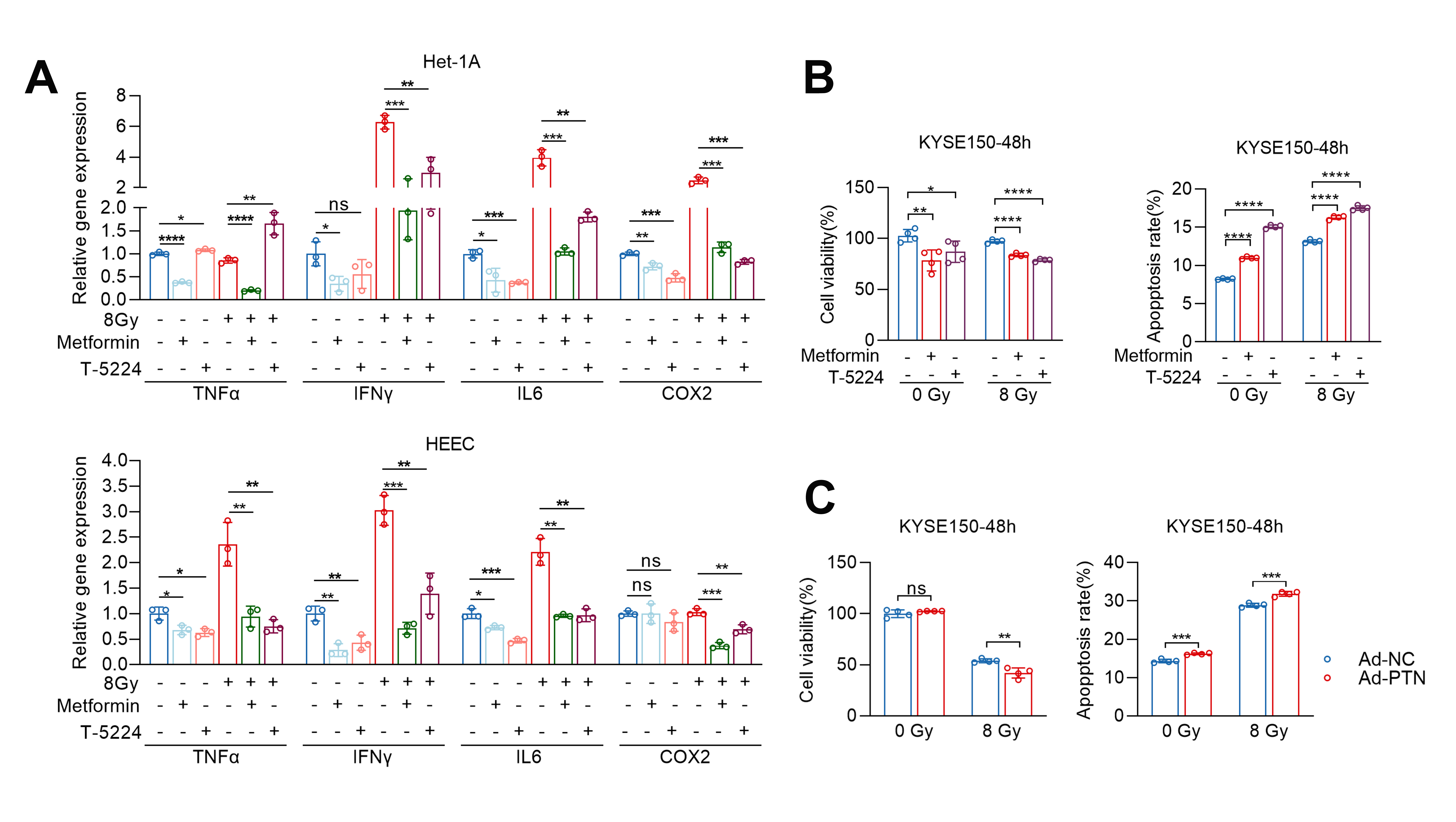
**

**Figure S8** Radiosensitizing effects of T-5224, metformin and PTN in human esophageal cancer cells. (A) mRNA levels of *TNFA*, *IFNG*, *IL6* and *PTGS2* in Het-1A and HEEC cells treated with T-5224 or metformin in 0 and 8 Gy X-ray radiation. (B) Cell viability and cell apoptosis assays of human esophageal cancer cells (KYSE150) treated with T-5224 or metformin in 0 and 8 Gy X-ray radiation. (C) Cell viability and cell apoptosis assays of KYSE150 cells infected with Ad-PTN and Ad-NC in 0 and 8 Gy X-ray radiation. Data are represented as mean ± SD and analyzed by an unpaired 2-tailed *t*-test (A, *n* = 3; B, C, *n* = 4). * *P* < 0.05; ** *P* < 0.01; *** *P* < 0.001; **** *P* < 0.0001; ns, not significant.

**
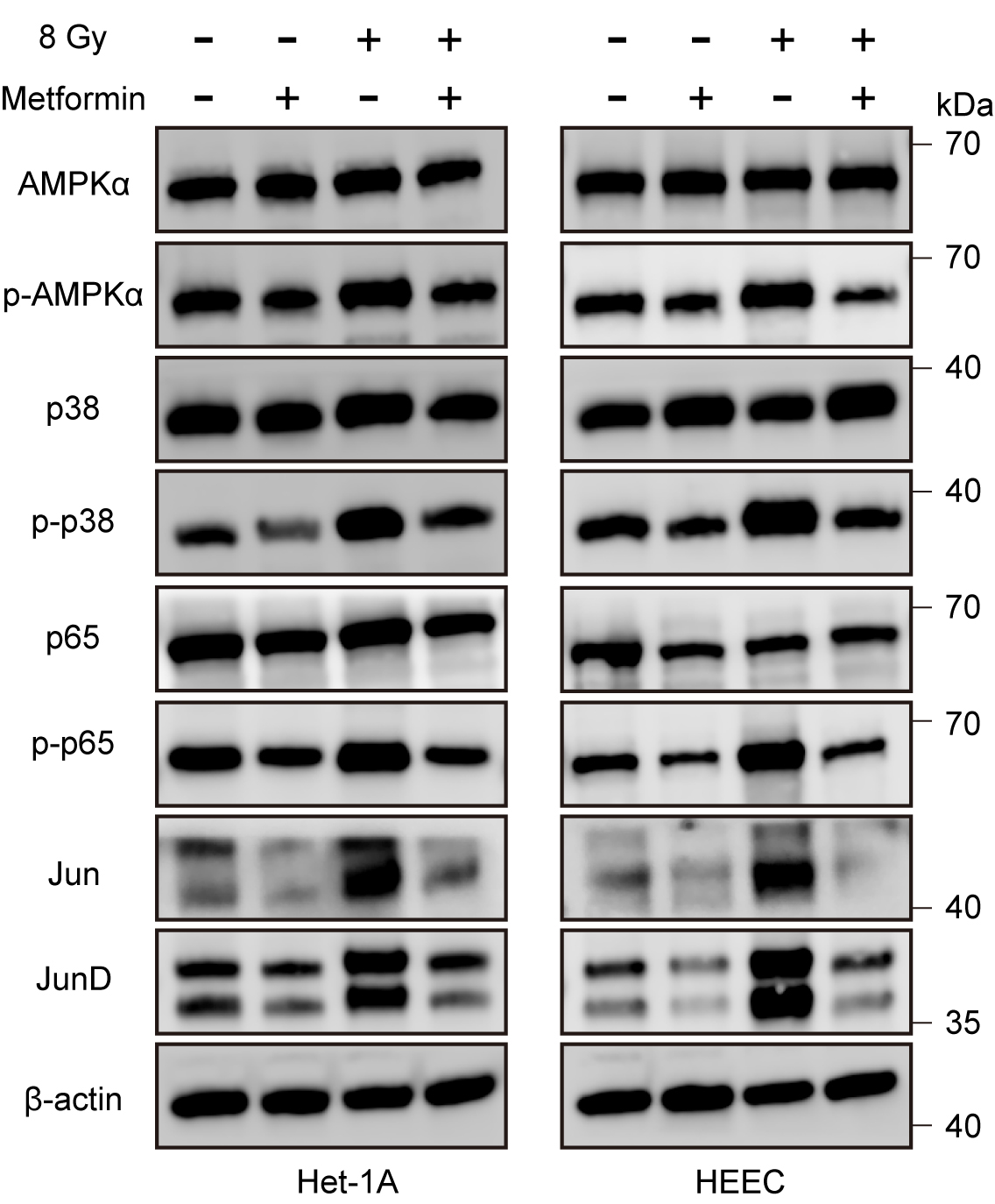
**

**Figure S9** Western blot analysis of AMPK/MAPK/NF-*κ*B/AP-1 axis proteins in Het-1A and HEEC cells treated with metformin in 0 and 8 Gy X-ray radiation. The proteins included AMPK*α*/p-AMPK*α* (AMPK), p38/p-p38 (MAPK), p65/p-p65 (NF-*κ*B), and Jun/JunD (AP-1).
